# DNA-mediated coupling of ATPase, translocase and nuclease activities of a Type ISP restriction-modification enzyme

**DOI:** 10.1093/nar/gkaa023

**Published:** 2020-01-24

**Authors:** Mahesh Kumar Chand, Vanessa Carle, K G Anuvind, Kayarat Saikrishnan

**Affiliations:** Division of Biology, Indian Institute of Science Education and Research, Pune 411008, India

## Abstract

Enzymes involved in nucleic acid transactions often have a helicase-like ATPase coordinating and driving their functional activities, but our understanding of the mechanistic details of their coordination is limited. For example, DNA cleavage by the antiphage defense system Type ISP restriction-modification enzyme requires convergence of two such enzymes that are actively translocating on DNA powered by Superfamily 2 ATPases. The ATPase is activated when the enzyme recognizes a DNA target sequence. Here, we show that the activation is a two-stage process of partial ATPase stimulation upon recognition of the target sequence by the methyltransferase and the target recognition domains, and complete stimulation that additionally requires the DNA to interact with the ATPase domain. Mutagenesis revealed that a β-hairpin loop and motif V of the ATPase couples DNA translocation to ATP hydrolysis. Deletion of the loop inhibited translocation, while mutation of motif V slowed the rate of translocation. Both the mutations inhibited the double-strand (ds) DNA cleavage activity of the enzyme. However, a translocating motif V mutant cleaved dsDNA on encountering a translocating wild-type enzyme. Based on these results, we conclude that the ATPase-driven translocation not only brings two nucleases spatially close to catalyze dsDNA break, but that the rate of translocation influences dsDNA cleavage.

## INTRODUCTION

Helicases and translocases are the primary motors facilitating nucleic acids transactions in a cell (1). They hydrolyze nucleoside triphosphate and use the chemical energy to perform mechanical work. Helicases unwind double stranded (ds) DNA and directionally move (translocate) along the unwound single stranded DNA, while translocases translocate along dsDNA (1). The motor is often part of a multidomain and multifunctional protein and orchestrates its activity with those of the other functional domains to accomplish a specific task. The detailed mechanism of how the different domains are functionally coupled to the motor in many of these molecular machines is not well understood (1,2). Here, we have studied the ATPase activity of the Type ISP restriction-modification (RM) enzyme leading to translocation initiation, and gain insights into the importance of enzyme translocation for its single-strand nicking and double-strand DNA cleavage activities.

**Figure 1.**
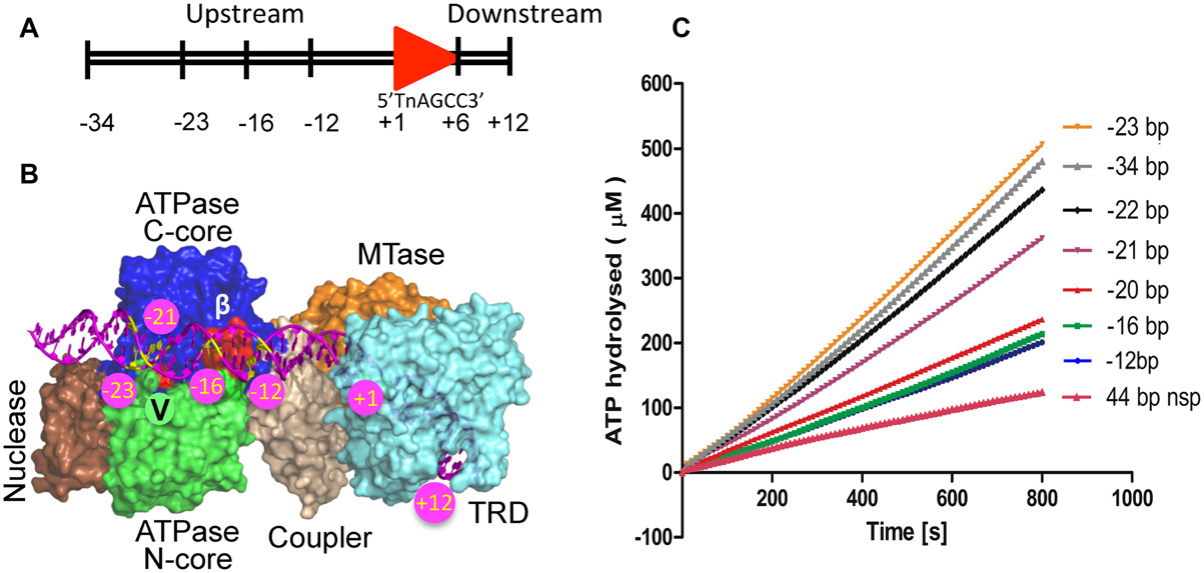
DNA length-dependent stimulation of ATPase activity. (**A**) The scheme used for numbering the base pairs in a DNA substrate (shown here is a 46 bp specific DNA). The red arrowhead represents the target sequence. The target sequence of LlaBIII is written below the arrowhead. (**B**) Model of LlaBIII bound to DNA (magenta). The yellow base pairs show the position of the upstream end of some of the substrates used for the study. The β-hairpin loop and Arg564 of motif V are colored in red (see text for details). (**C**) Rate of ATP hydrolysis by WT LlaBIII in presence of specific DNA with varying length upstream of the target sequence. The plot also shows the amount of ATP hydrolyzed in presence of non-specific (nsp) DNA. The assay was performed with 50 nM LlaBIII, 500 nM DNA and 1 mM ATP. Shown are the averages of three replicates.

RM enzymes are bacterial defense against invading foreign DNA and preventing infection by bacteriophages. Type ISP RM enzymes are made of a single polypeptide chain comprising four functional domains - a nuclease, a motor belonging to the Superfamily 2 helicase-like ATPase, an N6-adenine methyltransferase (MTase) and a target recognition domain (TRD) (3). The MTase methylates the target adenine base (position +1 in Figure 1A) of the target sequence in presence of cofactor AdoMet. The nuclease cleaves unmodified DNA only in presence of ATP (3). Methylation allows the enzyme to discriminate between the modified host genomic DNA and the unmodified foreign DNA. Unmodified DNA is cleaved when two translocating Type ISP RM enzymes converge on the DNA in *cis* (4). DNA translocation powered by the ATPase is initiated only when it is bound to its unmodified target site (4,5). DNA cleavage requires the target sites of the two converging enzymes to be in head-to-head orientation (3,5–7).

Like many helicases whose ATPase activities are stimulated by DNA having specific structures, ATP hydrolysis by a Type ISP RM enzyme is activated by dsDNA containing its target sequence (4,5). The rate of ATP hydrolysis by the Type ISP RM enzyme is significantly higher in presence of specific DNA than in presence of non-specific DNA (5). The regulation of the ATPase activity by the target sequence is observed in the case of the other ATP-dependent RM enzymes, the Type I and Type III RM enzymes, too (8,9). The structure of the Type ISP RM enzymes LlaBIII and LlaGI bound to a DNA substrate mimic revealed that the target sequence is recognized by the TRD and MTase (4,10). The MTase is coupled to the ATPase by a coupler domain, and N-terminal to the ATPase is the nuclease domain. Furthermore, the structure revealed that at the target site the DNA is bent by ∼39° causing it to steer towards the ATPase.

The ATPase is physically >15 bp upstream to the target sequence anchored to the TRD and MTase (4). Previously, it has been shown that at least 23 bp upstream to the target sequence is required for LlaBIII to initiate translocation along the DNA (Figure 1A) (4). This prompted us to ask if the binding of the target sequence to the TRD and MTase is sufficient to stimulate the ATPase or, like in the case of translocation initiation, if a DNA long enough to interact with the ATPase is also required? It has been demonstrated in the case of some SF2 helicases that motifs III and V transduce the conformational changes arising from ATP hydrolysis to DNA translocation (11–18). Using mutagenesis, we also sought to find if motifs III and V play a role in coupling the two activities in Type ISP RM enzymes.

Translocation of the Type ISP RM enzyme along the DNA is essential for nucleolytic cleavage (19). The convergence of two such translocating enzymes in a head-to-head direction brings the respective nucleases in proximity to form a collision complex. The collision complex nicks the top and the bottom strand of the DNA separated by ∼30 bp. Movement and stalling of the ATPase-active collision complex formed of a pair or multiple Type ISP RM enzymes result in further nicks leading to dsDNA break (4,20). Convergence of the nucleases from two enzymes is a requirement for DNA cleavage by the ATP-dependent Type I (21–22) and Type III RM enzymes (9), too. It is, however, not clear if the sole purpose of DNA translocation is to bring the nucleases close in space, or if the rate of translocation with which the enzymes collide also contribute to the activation of the nucleases.

It has been hypothesized that the collision of the converging enzymes alters the conformation of the Type ISP RM enzymes and activates their nuclease (20). Through the biochemical characterization of the wild-type (WT) and mutant enzymes designed to study the coupling of the ATPase to the other functional activities, we demonstrate that both single-strand nicking and dsDNA cleavage not only require an active translocase but is also dependent on the rate of translocation. The study reported here provides new insights into the role of substrate DNA in coupling target sequence recognition to ATPase and translocase activities, and the importance of the ATPase activity for the functioning of a Type ISP RM enzyme.

## MATERIALS AND METHODS

### DNA

Site directed mutagenesis was carried out and the ATPase domain mutants (LlaBIII^K385A^, LlaBIII^ΔLoop^, LlaBIII^PolyALA^, LlaBIII^T376A^ and LlaBIII^R564A^) were cloned into pRSF vector under NcoI and XhoI sites using forward LB-pRSF-F and reverse primer LB-pRSF-R alongwith mutation site specific primers K385A-R, ΔLoop-2G-R, ΔLoop-2G-F, PolyALA-R, T376A-R and R564A-R, respectively. The sequences of all the primers used are listed in Supplementary Table S1. The gene sequences of the mutants were confirmed by DNA sequencing. A 1085 bp long DNA substrate having two target sites of LlaBIII in head-to-head orientation was generated by PCR using LlaBIII gene as template with forward primer LB-1085-F and reverse primer LB-1085-R. 1758 bp and 2551 bp DNA substrate for cooperation assay were amplified in a similar manner using forward and reverse primers LJ1HISF and LJ1HISR1, and 200 bp_FP and 1439 bp_FP, respectively. All the DNA oligomers used as primers and as substrates for the ATPase assays were purchased from Sigma/Integrated DNA Technologies.

### Purification of LlaBIII

LlaBIII and its mutants were purified as described earlier (4).

### DNA cleavage assay

A 1085 bp long DNA with two LlaBIII target sites in head-to-head orientation separated by 155 bp was used as the substrate for nucleolytic DNA cleavage assay. The enzyme (WT LlaBIII or mutants) and DNA were mixed in TMDK buffer (50 mM Tris–Cl pH 8.0, 10 mM MgCl_2_, 1 mM DTT, 150 mM KCl) on ice and the reaction was started by adding 4 mM ATP (19). The reaction mix was incubated for 30 min at 25°C. Reactions were stopped by addition of half the volume of STEB (0.1 M Tris–Cl pH 7.5, 0.2 M EDTA, 40% w/v sucrose, 0.4 mg/ml bromophenol blue, 0.5% SDS) and analyzed on 1% agarose gel. In the case of concentration dependent assays, the same reaction mixture was used and only the concentration of the enzyme was varied.

LlaBIII and LlaGI can cooperate and cleave the substrate DNA containing one LlaBIII and one LlaGI site (19). For cooperation assay, a 1758 bp/2551 bp long DNA containing a LlaBIII site in head-to-head orientation with a LlaGI site was used. 250 nM LlaBIII or its mutant and LlaGI were incubated with 10 nM DNA for 5 min in TMDN buffer (50 mM Tris–Cl pH 8.0, 10 mM MgCl_2_, 1 mM DTT, 50 mM NaCl) at 4°C and the reaction was started by addition of 4 mM ATP. The reaction mixes were incubated for 30 min at 25°C and were stopped by addition of half the volume of STEB and analyzed on 1% agarose gel. The positions of cleaved DNA products were determined using GelQuant Express software (Life Technologies). The densitometric plots were obtained using ImageJ software.

### DNA nicking activity

A 1085 bp long DNA with two LlaBIII target sites in head-to-head orientation separated by 155 bp was used as the substrate for DNA nicking assay. Varying concentrations (50, 100, 200 and 500 nM) of LlaBIII or LlaBIII^R564A^ was mixed with the DNA and the reaction was started with addition of 4 mM ATP followed by 30 minutes incubation at 25°C. Reaction was stopped by adding equal volume of 2× formamide gel loading dye (95% v/v formamide, 0.025% SDS, 0.5 mM EDTA and 0.03% bromophenol blue). The reaction mixture was heated at 95°C for 10 min and the nicked DNA products were resolved on 8% urea–formamide polyacrylamide gel electrophoresis (PAGE). The densitometry plots were prepared using ImageJ software.

### Circular dichorism spectroscopy and nanoDSF

Circular dichroism (CD) spectra of LlaBIII and its mutants were recorded at 25°C using Jasco 815 spectrometer. 200 ul of 0.06 mg/ml protein samples in 20 mM phosphate buffer pH 8.0 with 100 mM KCl was added to 2 mm quartz cuvette and absorption spectra of the circularly polarized light were measured from 195 to 250 nm wavelength. Data was acquired at bandwidth 1 nm and scanning speed 100 nm/min. Sensitivity was kept high with five accumulations and data integration time (DIT) as 1 s. For accuracy, protein concentration was estimated by both Bradford assay and absorption measurement at 280 nm.

Temperature-dependent protein unfolding assay was carried out using NanoDSF (Prometheus NT.48, Nanotemper Technologies). 5 ul protein solution (1 mg/ml protein in buffer containing 50 mM Tris–Cl pH 8.0, 1 mM DTT and 100 mM KCl) was loaded in a capillary and the sample was heated from 20 to 95°C with a rate of 1°C/minute. Fluorescence intensity of protein was measured at 330 and 350 nm. The ratio of the fluorescence intensity at 330 and 350 nm, i.e. F350/F330, was plotted against the temperature gradient. The first derivative of F350/F330 was used to deduce the melting temperature.

### DNA binding assay

Binding assays were performed with 28 bp specific/non-specific DNA in binding buffer (50 mM Tris pH 8.0, 10 mM MgCl_2_, 1 mM DTT, 150 mM KCl). 250 nM DNA was allowed to bind with varying concentrations of LlaBIII or LlaBIII^ΔLoop^ (0, 10, 50, 100, 250, 500, 1000, 2500, 5000 nM). Protein and DNA were mixed and incubated on ice for 10 min. Reactions were stopped by adding half the volume of STB (0.1 M Tris pH 7.5, 40% w/v sucrose, 0.4 mg/ml bromophenol blue) and loaded on 6% native PAGE run at 4°C. The gel was stained with ethidium bromide and imaged on Typhoon TRIO+ variable mode imager (GE Healthcare).

### ATPase assay

NADH coupled ATPase assay was performed to compare the activity of LlaBIII and its mutants. Fluorescence intensities were measured using Varioskan Flash (Thermo Scientific). A 46 bp long DNA (Figure 1A) having one LlaBIII target site was used as a specific substrate and a 40 bp long DNA with no target sequence was used as non-specific DNA. 50 nM of WT or mutant enzyme was incubated with 500 nM of specific or non-specific DNA for 5 min. ATP was added and reaction was shaken for 30 s before the measurements were taken. Absorbance at 340 nm was recorded every 10 s for 800 s at 25°C. ADP standard was performed for each set of experiment. The concentration of ATP hydrolyzed was calculated at each time interval using a line equation *Y* = *mX* + *C* (where *Y* = absorbance, *m* = slope, *X* = concentration of ADP produced or ATP hydrolyzed and *C* = intercept on Y axis) obtained from standard plot with different ADP concentrations.

The assay measured steady-state ATPase activity, with the enzyme running off the DNA and rebinding. Kinetics parameters *V*_max_, *K*_m_ and *k*_cat_ were obtained by ATP concentration-dependent assay with ATP concentrations of 0.25, 1, 4, 16, 64, 128, 256, 512, 1024 and 2048 μM. The data with ATP hydrolysis rates plotted against different ATP concentration were fit to Michaelis–Menten equation using GraphPad PRISM 5.

### Triplex DNA and triplex displacement assay

Triplex forming oligonucleotide (TFO_1 and TFO_3) was radiolabelled with T4 Polynucleotide Kinase (New England Biolabs Inc.) and ATPγ^32^P at 37°C for 30 min. Labeled TFOs were purified by mini-quick spin oligo (Roche, Germany) column and kept at −30°C till further use. 50 nM pONE plasmid with single site for LlaBIII (6) was linearized with HindIII. 25 nM of TFOs were mixed with the linearized plasmid in MM Buffer (10 mM MES pH 5.5 and 25 mM MgCl_2_) and incubated at 57°C for 15 min in two separate vials. The reaction mix was further incubated at 20°C overnight to allow the triplex DNA formation (23). Triplex DNA was stored at −20°C until further use.

50 nM enzyme and 1 nM triplex DNA was used in TMDK buffer (50 mM Tris pH 8.0, 10 mM MgCl_2_, 1 mM DTT and 150 mM KCl). The enzyme was incubated with triplex DNA in TMDK buffer for 5 min to allow binding to the target site. The reaction was initiated by addition of 4 mM ATP and incubated for 10 min at 20°C. The reaction mixture also contained unlabeled TFO to prevent non-specific binding of displaced TFO to LlaBIII. In case of time-dependent triplex assay, the incubation time of the reaction mixture was varied. Half the volume of GSMB (15% w/v glucose, 3% w/v SDS, 250 mM MOPS pH 5.5 and 0.4 mg/ml bromophenol blue) was added to stop the reaction and analyzed on 6% Native PAGE (acrylamide:bisacrylamide 29:1) prepared in 40 mM Tris-acetate, 5 mM Na-acetate, 5 mM MgCl_2_, 0.1 mM EDTA pH 5.5 at 4°C. The gel was dried in Gel dryer model 583 attached with HydroTech™ Vaccum Pump supplied by BioRad at 80°C for 1$\frac{1}{2}$ h. The dried gels were exposed to phosphorimager plate (GE Healthcare) overnight. Gels were imaged on Typhoon TRIO+ variable mode imager (GE Healthcare), and the intensities of the bands were quantified using ImageJ software. All the graphs were plotted in GraphPad PRISM 5. The intensities for the enzyme and triplex DNA complex were considered as 100%, and the displacement was calculated for the lanes where ATP was added. In the time-dependent experiment, the intensities for zero time point was considered as 100% and the intensities for all time points were normalized. For lag time calculation, we fitted the triplex data to second exponential equation.

$$\begin{equation*}y\ = {A_1}.\left( {1 - {{\rm exp}^{ - {k_1}\left( {T - {T_{{\rm lag}}}} \right)}}} \right) + {A_2}.\left( {1 - {{\rm exp}^{ - {k_2}\left( {T - {T_{{\rm lag}}}} \right)}}} \right)\end{equation*}$$

where *y* is percentage of triplex displaced. *A*_1_ & *A*_2_ are amplitudes of the phases and *k*_1_ & *k*_2_ are the rates of the two phases. *T* is the time of incubation after ATP mixing and *T*_lag_ is the lag time or time required by the enzyme to start triplex displacement.

## RESULTS

### ATPase activation is a two-stage process dependent on DNA length

To find if DNA target recognition is sufficient to stimulate the LlaBIII ATPase, we measured the ATPase activity of the enzyme in presence of a 22 bp DNA having the target sequence. As defined previously (4), each base pair (bp) in the substrate DNA is numbered such that the target adenine for methylation and its complementary base is +1, the base pairs upstream (towards the ATPase domain) of the target sequence have a negative numbering and those downstream have a positive numbering (Figure 1A and B). The 22 bp DNA had 4 bp (+10) downstream of the target sequence and 12 bp (–12) upstream. This DNA was not long enough to interact with the ATPase. In comparison to a non-specific DNA, the 22 bp DNA stimulated the ATPase activity by ∼2-fold (Figure 1C).

Increasing the length upstream of the target site in the direction of the ATPase (Figure 1B) from –12 to –20 did not affect the ATPase activity (Figure 1C). However, an upstream length longer than 20 bp increased the ATPase activity by ∼2-fold (Figure 1C). The clustering of the ATPase activity into two distinct sets with one having a higher ATP hydrolysis rate than the other beyond a threshold length suggested that there are two stages in achieving complete activation of the ATPase. The first stage involves the binding of the target sequence to the MTase and TRD. This stage does not require the DNA to be long enough to engage with the ATPase motor (Figure 1B). The second stage requires a DNA long enough (>–20) to interact with the ATPase (Figure 1B).

This is consistent with our previous observation that DNA translocation by LlaBIII, which is driven by ATP hydrolysis, is dependent on the upstream length of the specific DNA (4). Interestingly, though, activation of the ATPase requires at least 21 bp upstream of the target sequence (Figure 1C), translocation initiation requires at least 23 bp (4). This implies that full activation of the ATPase is not sufficient for DNA translocation to initiate, and the latter requires an additional 2 bp upstream.

### β-Hairpin loop of the ATPase N-core is essential for nucleolytic activity

**Figure 2.**
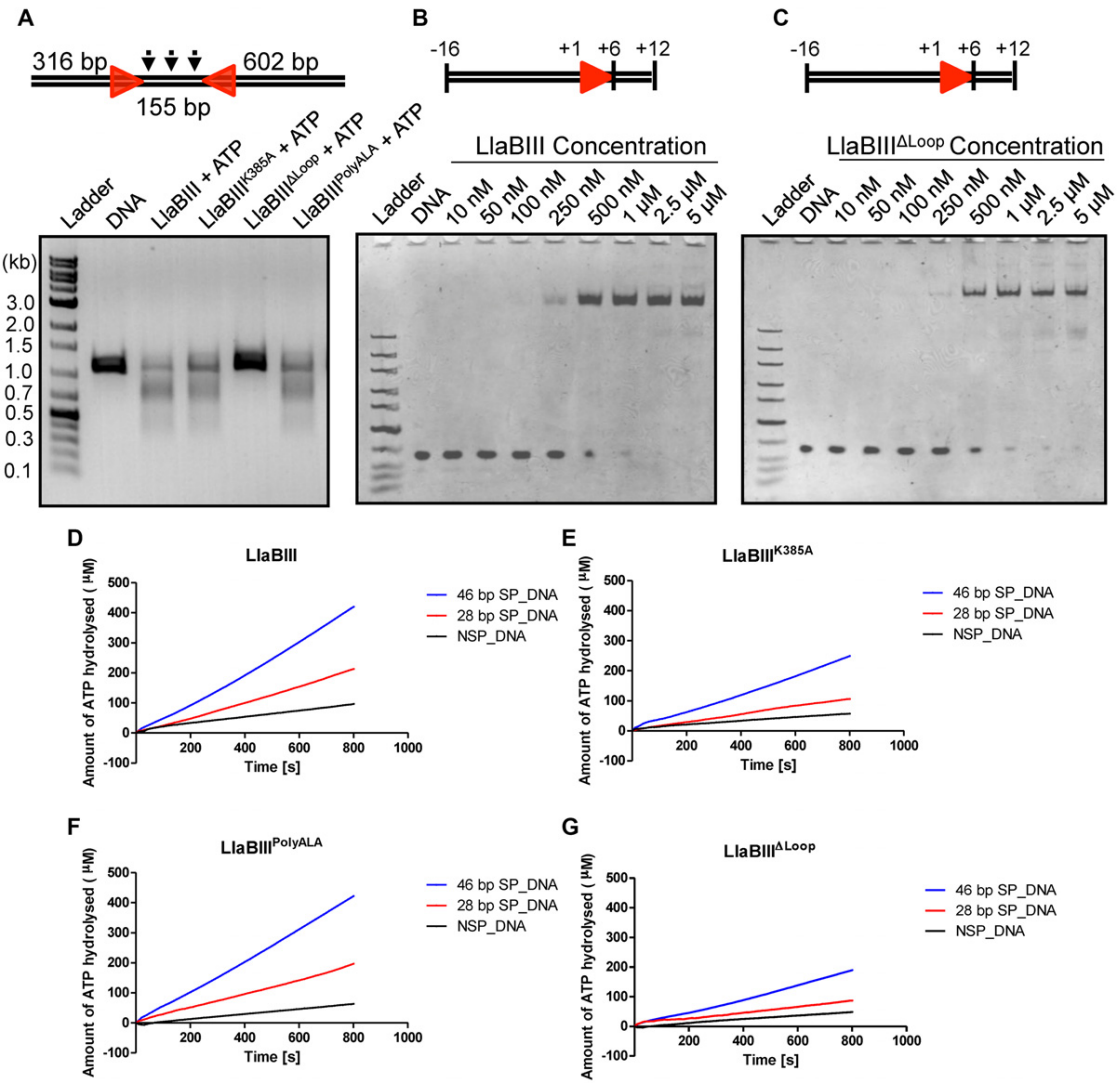
Functional characterization of β-hairpin loop mutants of LlaBIII. (**A**) Results of the DNA cleavage assay showing the nucleolytic activity of LlaBIII and its β-hairpin loop mutants (LlaBIII^K385A^, LlaBIII^ΔLoop^ and LlaBIII^PolyALA^) on 1085 bp DNA with head-to-head oriented sites. (**B**, **C**) Electrophoretic mobility shift assays comparing the DNA binding affinities of WT LlaBIII and LlaBIII^ΔLoop^ for a specific 28 bp DNA. (D–G) Comparison of the ATPase activities of (**D**) LlaBIII, (**E**) LlaBIII^K385A^, (**F**) LlaBIII^PolyALA^ and (**G**) LlaBIII^ΔLoop^ in presence of 46 and 28 bp specific DNA (SP_DNA) and a 44 bp non-specific DNA (NSP_DNA). 50 nM LlaBIII or its mutants, 500 nM DNA and 1 mM ATP were used for the NADH coupled ATPase assay carried out at 25°C. Shown are the averages of three replicates.

The analysis in the previous section reveals that full activation of the ATPase requires not only DNA target recognition but also engagement of the DNA with the ATPase domain. The first structural element of the translocating ATPase that contacts the DNA is a β-hairpin loop in its *N*-core (Figure 1B) (4,10). This hairpin loop interacts with the phosphodiester backbone of the DNA through bonds formed by the main chain amino group of K389 and D390. We had earlier proposed that this region might be involved in paddling the DNA during translocation (4). To find if the loop was important for LlaBIII activities, we mutated the loop. We made three sets of mutants—LlaBIII^K385A^ in which a highly conserved lysine at position 385 (Supplementary Figure S1) was mutated to alanine; LlaBIII^PolyALA^ in which all the loop residues from 383 to 389 were mutated to alanine; and LlaBIII^ΔLoop^ in which residues 383–390 were replaced with two glycines. In one of the monomers of the LlaBIII-DNA structure, the side chain of Lys385 is within hydrogen bonding distance of N6 of an adenine base (4). Nucleolytic assay revealed that LlaBIII^ΔLoop^ failed to cleave DNA (Figure 2A). In contrast, LlaBIII^K385A^ and LlaBIII^PolyALA^ had nucleolytic activities comparable to that of the WT enzyme (Figure 2A). Comparison of the secondary and tertiary structural profile of the WT and the mutant enzymes using circular dichroism and nanoDSF indicated that the mutations did not affect the structural integrity of LlaBIII (Supplementary Figure S2A and B).

To find why LlaBIII^ΔLoop^ failed to cleave DNA, we tested the affinity of LlaBIII^ΔLoop^ for specific DNA. The mutant and the WT enzyme bound to DNA equally well and could discriminate between specific and non-specific DNA (Figure 2B and C, Supplementary Figure S3). Increasing the concentration of LlaBIII^ΔLoop^ also failed to elicit DNA cleavage (Supplementary Figure S4). Next, we compared the ATPase activities of WT LlaBIII and LlaBIII^ΔLoop^. The ATPase activities of LlaBIII^K385A^ and LlaBIII^PolyALA^, which have nucleolytic activities comparable to the WT enzyme, were also measured. The rate of ATP hydrolysis was measured in presence of either a specific or a non-specific DNA. The ATPase activities of WT and the mutant enzymes were significantly higher in the presence of specific DNA than non-specific DNA (Figure 2D–G), implying that, despite the mutations of the β-hairpin loop, the ATPase activity was stimulated by specific DNA. Though specific DNA stimulated the ATPase activities of the four enzymes, they showed different rates of ATP hydrolysis (Figure 2D–G). WT and LlaBIII^PolyALA^ had similar ATPase activities. However, LlaBIII^K385A^ and LlaBIII^ΔLoop^ had relatively lower ATPase activities.

LlaBIII-Lys385 is close enough to interact with the DNA *via* its side chain (4), and, as a consequence its mutation, modulates the stimulation of the ATPase by the substrate DNA. Interestingly, replacement of all the loop residues with alanine (LlaBIII^PolyALA^) overcame the decrease in the ATPase due to K385A mutation (Figure 2E and F). As the nucleolytic activity of LlaBIII^K385A^ was found to be comparable to the WT enzyme, we know that the lower ATPase activity of LlaBIII^K385A^ is sufficient for efficient DNA cleavage to occur. Also, both LlaBIII^K385A^ and LlaBIII^ΔLoop^ displayed the DNA length-dependent two-stage ATPase activation implying that the mutations did not affect the mode of activation (Figure 2E and G). As LlaBIII^K385A^ and LlaBIII^PolyALA^ were nucleolytically active while LlaBIII^ΔLoop^ was inactive, we concluded that the side chains of the residues constituting the β-hairpin loop were not important for the activity, while the interactions made by the main chains atoms of residues 389 and 390 with the DNA were important.

### Deletion of the β-hairpin loop affects DNA translocation

**Figure 3.**
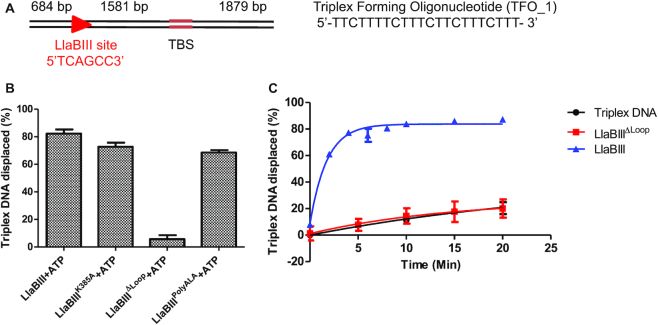
(**A**) The triplex DNA substrate used for DNA translocation assay. Red arrow shows the LlaBIII target site and direction of translocation. TBS is the TFO binding site. (**B**) Bar graph showing ATP-dependent triplex displacement by LlaBIII, LlaBIII^K385A^, LlaBIII^PolyALA^ and LlaBIII^ΔLoop^ in 10 min at 20°C. 4 mM ATP was used in the reaction. Shown are the averages of three replicates. (**C**) Time dependent triplex displacement assay showing that LlaBIII^ΔLoop^ is an inactive translocase. Shown are the averages of three replicates.

The biochemical analysis of LlaBIII^ΔLoop^ demonstrated that the mutant enzyme is proficient in DNA binding and ATP hydrolysis, but it does not produce dsDNA break. dsDNA cleavage by LlaBIII requires two translocating enzymes to converge on the DNA. Hence, we sought to find if LlaBIII^ΔLoop^ is active as a translocase. Toward this, we used a triplex displacement assay that has previously been used to study the translocation activity of LlaBIII (6). The assay used a ∼4.2 kb long linear DNA having a single LlaBIII target site located 1581 bp upstream of a triplex binding site, which was bound to a 22 nt long oligo to form the triplex (Figure 3A). As expected, the WT enzyme was an active translocase, which displaced almost ∼83% of the TFO from the substrate DNA in 10 min.

LlaBIII^K385A^ and LlaBIII^PolyALA^, which are active nucleases, displaced ∼73% and ∼69% of the triplex oligo. Interestingly, the decreased ATPase activity of LlaBIII^K385A^ did not affect its translocase activity as much as that of LlaBIII^ΔLoop^, which did not displace the triplex oligo (Figure 3B, Supplementary Figure S5). Time-dependent triplex displacement assay showed that LlaBIII displaces ∼61% and 83% of triplex DNA in 2 and 4 min respectively whereas LlaBIII^ΔLoop^ did not displace the triplex even after 20 min of incubation (Figure 3C, Supplementary Figure S6). This study clearly demonstrated that the β-hairpin loop is important for DNA translocation by LlaBIII, and deletion of the loop affected the nuclease activity because of deficiency in translocation. The role of the β-hairpin loop in translocation is consistent with the earlier proposal that this structural element might be involved in paddling the DNA (4). As LlaBIII^ΔLoop^ is an active ATPase but an inactive translocase, we concluded that the β-hairpin loop contributes to coupling the two activities.

### Effect of mutation of SF2 helicase motifs III and V on LlaBIII activities

In SF2 helicase-like ATPases, motifs III and V are attributed to couple ATPase and translocase activities (11–18). In the previous section, we demonstrated that the β-hairpin loop in LlaBIII couples the two activities. A structural model of LlaBIII bound to longer DNA suggested that the presence of the β-hairpin loop hides the proximal motif III from interacting with the DNA, while motif V was positioned to interact with the DNA. In SF2 ATPases where the role of motif III in substrate binding and/or coupling of the ATPase to the translocase activity has been demonstrated, the substrate is found to interact with motif III (13). This led us to compare the significance of motifs III and V for the LlaBIII activities.

**Figure 4.**
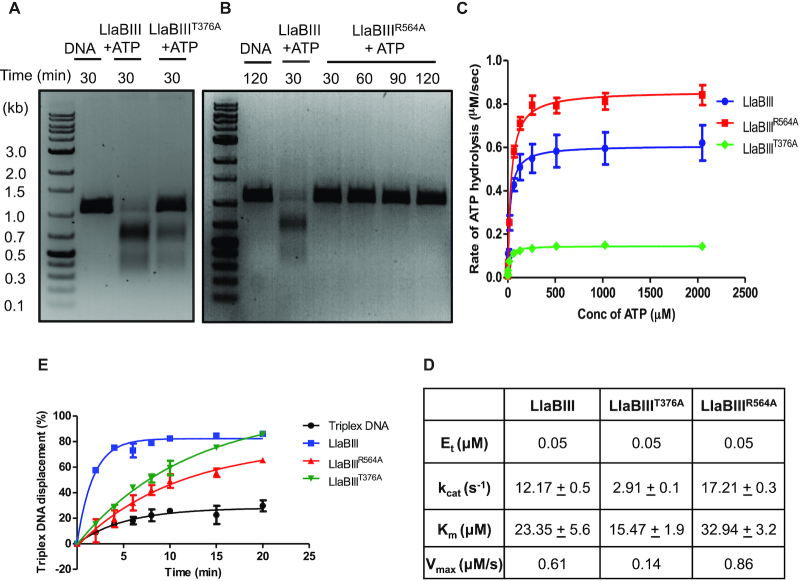
Biochemical characterization of motifs III and V of LlaBIII ATPase domain. (**A, B**) DNA cleavage by LlaBIII^T376A^ and LlaBIII^R564A^. In comparison to WT LlaBIII, LlaBIII^T376A^ shows less DNA cleavage activity while the motif V mutant LlaBIII^R564A^ is nucleolytically inactive. (**C**) The kinetics of ATP hydrolysis by WT LlaBIII (blue), LlaBIII^T376A^ (green) and LlaBIII^R564A^ (red). 50 nM LlaBIII or the mutants with 500 nM DNA concentration were used in NADH coupled ATPase assay. The ATP concentrations used were 0.25, 1, 4, 16, 64, 128, 256, 512, 1024 and 2048 μM. Reactions were carried out at 25°C. Shown are the averages of three replicates. (**D**) Kinetic parameters for ATP hydrolysis obtained from (C). (**E**) Triplex displacement assay showing the percentage of triplex DNA displaced by LlaBIII (blue), LlaBIII^T376A^ (green) and LlaBIII^R564A^ (red) over different time points (0, 2, 4, 6, 8, 10, 15 and 20 min) at 20°C. Black line is triplex DNA control showing the dissociation of TFO from the DNA in the absence of protein over the same time points. The reaction was performed with 4 mM ATP concentration. Shown are the averages of three replicates.

We mutated Thr376 and Arg564, the most conserved residues of motifs III and V, respectively, to alanine. LlaBIII^T376A^ cleaved the substrate DNA, but in comparison to the WT enzyme the activity was noticeably weak (Figure 4A). In contrast, the motif V mutant LlaBIII^R564A^ showed little dsDNA cleavage activity even at a longer reaction time of 120 minutes (Figure 4B). We proceeded to measure the ATPase activity of LlaBIII^T376A^, which was found to be ∼4-fold lower than WT LlaBIII, and about 2- and 1.5-fold lower than LlaBIII^K385A^ and LlaBIII^ΔLoop^, respectively (Figure 4C and D, Supplementary Figure S7). LlaBIII^R564A^, on the other hand, displayed ATPase activity comparable to the WT enzyme (Figure 4C and D). Despite this, LlaBIII^R564A^ could not cleave the substrate DNA even at high enzyme concentration of 4 μM (Supplementary Figure S8).

We proceeded to compare the translocase activities of LlaBIII^T376A^ and LlaBIII^R564A^ with WT LlaBIII using triplex displacement assay as described above. In comparison to WT LlaBIII, LlaBIII^R564A^ took longer time to displace the triplex than LlaBIII^T376A^. WT LlaBIII was able to displace ∼77% of the triplex DNA in 4 min, while LlaBIII^T376A^ displaced a similar amount (∼70%) in 10 minutes and LlaBIII^R564A^ took 20 min to displace ∼67% of the triplex DNA (Figure 4E, Supplementary Figure S9). This was indicative of reduction in the rate of DNA translocation by LlaBIII^T376A^ and LlaBIII^R564A^(24). Interestingly, while LlaBIII^T376A^ displayed a visible though a less efficient nucleolytic activity, R564A mutation had negligible nuclease activity (Figure 4B).

**Figure 5.**
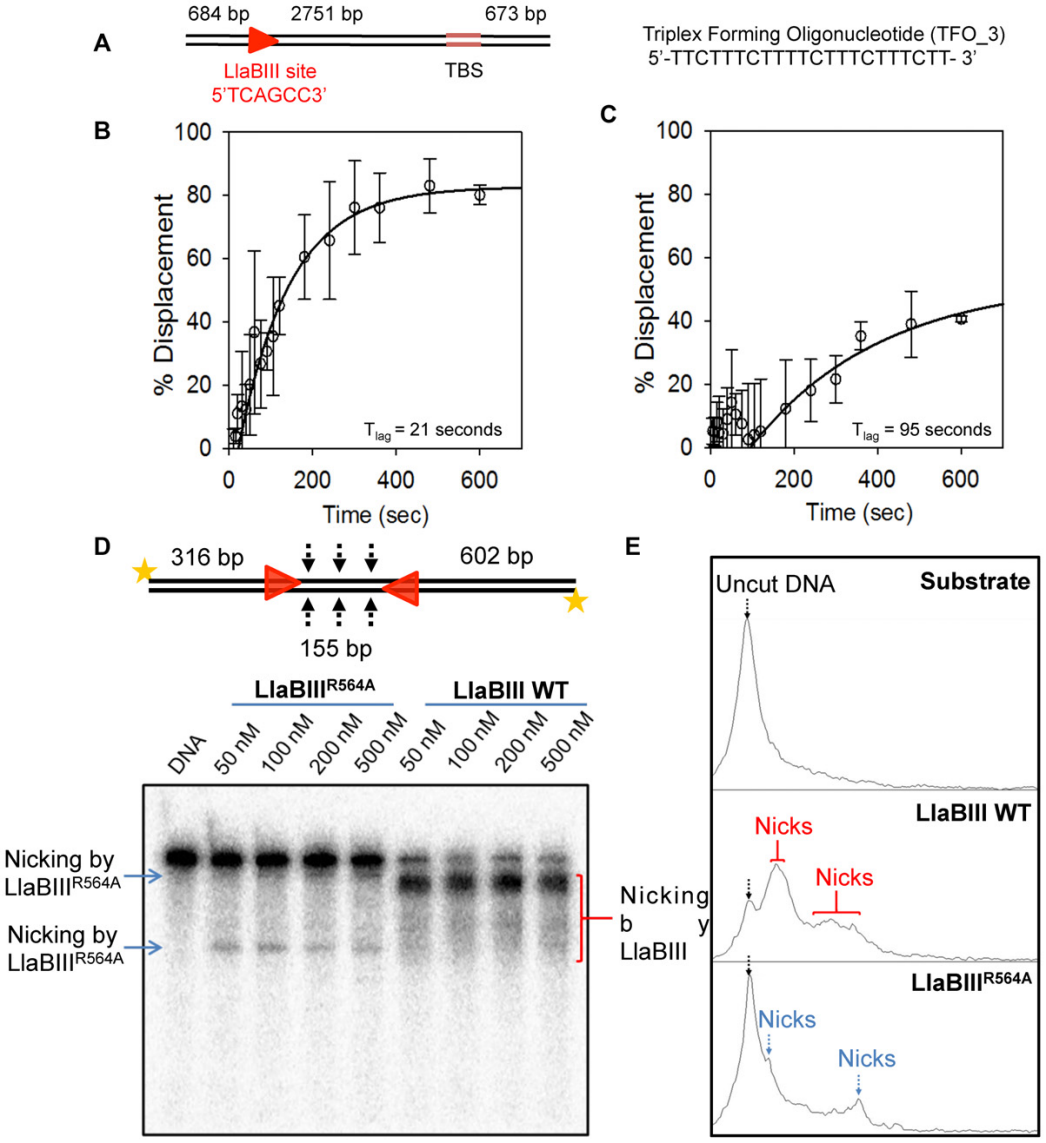
DNA translocation and nicking activity of LlaBIII and LlaBIII^R564A^. (**A**) Triplex DNA substrate with 2751 bp separation between LlaBIII target sequence and TFO binding site (TBS). Time dependent triplex displacement assay for (**B**) LlaBIII and (**C**) LlaBIII^R564A^ using the above substrate. Hollow circles with error bars shows percentage displacement at different time points. Black solid line shows double exponential fit used to calculate the lag time (*T*_lag_) where data points after 120 seconds were used for the fit. Different time points at which data was collected are 0, 5, 10, 15, 20, 30, 40, 50, 60, 75, 90, 105, 120, 180, 240, 300, 360, 480 and 600 s. (**D**) Denaturing urea-formamide PAGE showing concentration dependent DNA nicking by LlaBIII and LlaBIII^R564A^. 2 nM 1085 bp long 5′-end radiolabeled DNA substrate with a pair of head-to-head oriented target site was used. (**E**) Densitometry analysis of the nicked products by LlaBIII and LlaBIII^R564A^ in the PAGE in (D). Black arrow shows the position of the intact DNA (uncut DNA). Red arrows marks the peaks obtained as a result of nicking by LlaBIII, whereas blue arrows marks the positions of the nick product from the LlaBIII^R564A^. 4 mM ATP was used in the reaction.

Additionally, the triplex displacement of radiolabeled TFO by LlaBIII or LlaBIII^R564A^ was measured from 5 to 600 s by manually stopping the reaction. To improve the accuracy of measurement at lower time points, we increased the separation between the target sequence and triplex binding site to 2751 bp (Figure 5A) instead of 1581 bp used in previous experiments (Figure 4E). Percentage triplex displaced was plotted against time and fitted to a double exponential equation (see Materials and Methods). The intercept of the fit on the X-axis gave the lag time (23). Results from the experiments revealed that the lag time for triplex displacement by LlaBIII was 21 s whereas the lag time for LlaBIII^R564A^ was 95 s (Figure 5B and C), under the experimental conditions used. Hence, we concluded that LlaBIII^R564A^ is an inefficient translocase, and that the inefficient translocation affected the dsDNA cleavage activity of LlaBIII^R564A^.

The inefficient translocation of LlaBIII^R564A^ could be a result slower initiation of translocation, which possibly involves remodeling of TRD, MTase and target site to release the target-bound enzyme to facilitate the movement; and/or lower processivity of translocating LlaBIII^R564A^; and/or slower rate of translocation of LlaBIII^R564A^. If the mutation affected the release of the enzyme from target site, we should have observed dsDNA break over longer reaction time. Our assumption was based on the triplex displacement assay, which showed that LlaBIII^R564A^ leaves the target site and translocates to displace the TFO, but inefficiently (Figures 4E and 5C). Furthermore, LlaBIII^R564A^ could not form dsDNA breaks (Figure 4B). Hence, we concluded that, even if LlaBIII^R564A^ leaves the target site with a delay (we do not have evidence against this possibility), lack of dsDNA cleavage by this mutant was not a result of this delay.

If poor processivity was the primary cause affecting dsDNA cleavage by LlaBIII^R564A^, we would have expected dsDNA cleavage of at least a small fraction of DNA substrate having two target sequences in head-to-head orientation separated by 155 bp. Our expectation was based on the observation that LlaBIII^R564A^ reached the TFO binding site located either 1581 or 2751 bp away from the target sequence to displace the TFO in 10 min (Figures 4E and 5C). If processivity was the sole reason for lack of dsDNA cleavage, we would have expected a reasonable number of collision events to occur between LlaBIII^R564A^ molecules translocating from the target sequences separated by 155 bp forming dsDNA breaks. However, LlaBIII^R564A^ did not cleave the DNA substrate at all (Figure 4B). Consequently, we conclude that lower processivity is not the primary reason for the lack of dsDNA cleavage by LlaBIII^R564A^; rather it is due to slower rate of translocation.

To find if slower translocation affected single-strand nicking activity of LlaBIII^R564A^, the two-site DNA substrate with 5′ ends radiolabeled was incubated with LlaBIII^R564A^ and 4 mM ATP for 30 minutes and analyzed on 8% urea-formamide denaturing PAGE (Figure 5D). We noticed only a very small fraction of DNA was nicked (Figure 5D and E). This experiment was repeated three more times and similar results were obtained (Supplementary Figure S10). Furthermore, the small fraction of nicks formed did not result in any detectable dsDNA break (Figure 4B). Consequently, we concluded that the rate of translocation is important not only for dsDNA break, but also for activation of the nuclease to cause single-strand nicks.

### DNA cleavage by LlaBIII^R564A^ in cooperation with a wild-type enzyme

**Figure 6.**
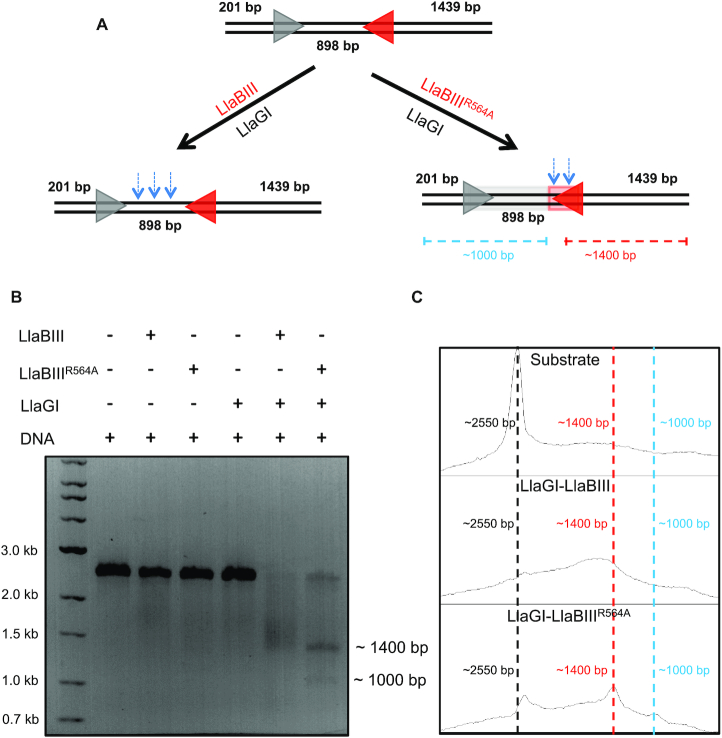
Heterologous cooperation assay. (**A**) DNA substrate used for the assay. The blue arrows illustrates the location of DNA cleavage by LlaGI-LlaBIII and LlaGI-LlaBIII^R564A^ based on the data in B and C. (**B**, **C**) DNA cleavage assay showing LlaGI-LlaBIII shreds the dsDNA, leading to a smear ranging from 1400 to 2200 bp whereas LlaGI-LlaBIII^R564A^ cleavage products are close to 1400 and 1000 bp. 10 nM DNA was incubated with 250 nM enzyme. The reaction was started by addition of 4 mM ATP.

Since LlaBIII^R564A^ was ATPase-competent but less efficient translocase, we hypothesized that the two converging LlaBIII^R564A^ did not converge with sufficient velocity to activate the nuclease. Consequently, we predicted that the collision of a slower LlaBIII^R564A^ with a faster WT enzyme might activate the nuclease to cleave DNA. To address this prediction, we used the homologue LlaGI, which recognizes a different recognition sequence (CTnGAYG). A 2551 bp long DNA containing a LlaBIII site and a LlaGI site in head-to-head orientation was used as the substrate (Figure 6A). As the DNA substrate had a single target site for the respective enzymes, WT LlaBIII, LlaBIII^R564A^ or LlaGI were unable to cleave the DNA on their own (Figure 6B).

In cooperation with LlaGI, however, WT LlaBIII and LlaBIII^R564A^ cleaved the DNA efficiently (Figure 6B). The DNA substrate was incubated with LlaGI and LlaBIII/ LlaBIII^R564A^ in presence of 4 mM ATP for 30 min and then analyzed visually and densitometrically on an agarose gel. The products of LlaGI-LlaBIII appeared as a broad smear (as expected for Type ISP RM enzymes) with median ranging around 1730 bp (Figure 6B and C). In contrast, the major cleavage product of LlaGI-LlaBIII^R564A^ appeared as a relatively sharp band of ∼1400 bp, which is close to the LlaBIII target sequence, and 1000 bp. This we interpreted as resulting from a collision of a very slow moving or a static site-bound LlaBIII^R564A^ with a fast moving LlaGI. This assay together with the lag time determined using triplex displacement assay provided strong evidence for decrease in the rate of translocation of LlaBIII^R564A^.

Similarly, LlaBIII^T376A^, which had a lower ATPase and dsDNA cleavage activities, displayed better nucleolytic activity in cooperation with LlaGI (Supplementary Figure S11). However, LlaBIII^ΔLoop^, which had an ATPase activity comparable to LlaBIII^T376A^ but little translocation activity, displayed negligible dsDNA cleavage in cooperation with LlaGI (Supplementary Figure S11). The results of these experiments were consistent with the above prediction that both efficiencies of single-strand nicking and dsDNA cleavage are dependent on the translocation rates of the colliding enzymes, and also highlighted that for DNA to be cleaved the Type ISP RM enzyme should have the ability to translocate DNA, even if inefficiently.

## DISCUSSION

The study described here aimed at understanding the mechanism of stimulation of the ATPase activity and its coupling to the other functional activities of the Type ISP RM enzyme. Like many other helicases that require substrates for stimulation of the ATPase, in the case of a Type ISP RM enzyme stimulation requires DNA containing a target sequence. Our results indicate that stimulation of the ATPase by specific DNA (containing a target sequence) is a two-stage process. The first stage happens on recognition of the target sequence by the TRD and MTase, resulting in stimulation of ATPase (Figure 1C).

**Figure 7.**
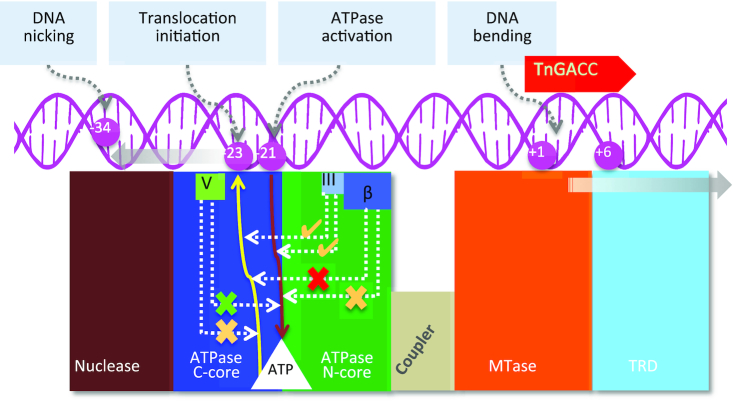
Schematic representation of DNA-mediated coupling of the ATPase to translocase and nuclease activities. The target sequence (+1 to +6) and the region of DNA bending upon target binding, which steers the DNA towards the ATPase domain, are shown. The continuous brown arrow from the DNA at -21 to the ATP binding pocket (white) indicates the length of DNA required for full stimulation of the ATPase, while the yellow line to the DNA at –23 indicates DNA length required for initiation of ATPase-coupled DNA translocation. The effect of the deletion of the β-hairpin loop, and mutation of motif III LlaBIII-Thr376 and motif V LlaBIII-Arg564 on the ATPase and translocase activities are illustrated using dotted arrow lines connecting the respective structural elements to the brown and yellow arrows illustrating DNA dependent ATPase and translocase activities. A tick mark indicates detectable nucleolytic cleavage, while a cross mark indicates no cleavage. An amber tick mark indicates a diminished ATPase or translocase but detectable nuclease activities, the green and the amber cross in the case of motif V indicates an active ATPase and a diminished translocase activities resulting in an inactive nuclease. The red and amber cross in the case of β-hairpin deletion mutant indicates reduced ATPase activity but inactive translocase and nuclease activities. The solid grey arrows indicate the direction of translocation of the DNA and the Type ISP RM enzyme, with respect to each other. -34 is the position of nicking by a static enzyme (20).

The ATPase activity is further stimulated (second stage) when a specific DNA with an upstream length >20 bp interacts with the ATPase domain. We further found that the shortest DNA length that can fully activate the ATPase is 22 bp, which however, is not long enough to initiate DNA translocation. Translocation initiation and DNA cleavage requires at least 23 bp upstream of the target sequence (4). This reveals that translocation initiation and DNA cleavage would not occur even if the ATPase were fully activated unless the DNA has the right length upstream of the target sequence (Figure 7). At the same time, DNA length and sequence requirements ensure that ATP hydrolysis is kept negligibly low unless the DNA has a target sequence and sufficient length upstream to initiate DNA translocation.

We speculate that interdomain movement between and intradomain conformational changes within the N-core and the C-core upon DNA binding would make the ATPase competent to hydrolyze ATP. This speculation is supported by structural changes seen in SF2 ATPases upon binding to their substrates (10,11,13,25–27). The observation that mere binding of the target sequence to the MTase and TRD stimulates the ATPase, though partially, suggests a direct allosteric communication between the target recognition unit (MTase-TRD) and the ATPase, possibly *via* the coupler domain. Complete stimulation is achieved *via* a substrate DNA long enough to interact with the ATPase. Determination and analysis of structures of Type ISP RM enzymes bound to DNA and ATP will be required to obtain the molecular basis of these conformational changes and allosteric communication.

Our study found the β-hairpin loop of the *N*-core of the ATPase, a structural element that the DNA interacts with, to be essential for nucleolytic cleavage as it coupled the ATPase to the translocase activity (Figure 7). The β-hairpin loop interacts with the DNA and facilitates translocation possibly by paddling the DNA resulting from the conformational changes in the ATPase domain upon nucleotide hydrolysis. The β-hairpin loop is not common to all SF2 helicase-like ATPases, and was first noticed in Type ISP RM enzymes (4). A structure and sequence analyses of Type I RM enzymes revealed that some of these enzymes have a loop at an equivalent position, and that they may perform a function similar to that of the β-hairpin loop of Type ISP RM enzymes (Supplementary Figure S12).

The conserved Arg564 of motif V in Type ISP RM enzyme LlaBIII also contributes to coupling the ATP hydrolysis to the translocase activity (Figure 7). Interestingly, we observed slightly higher ATP hydrolysis rate in case of LlaBIII^R564A^ than LlaBIII. This is reminiscent of the mutation of coupler motif III residues in eIF-4A, which leads to loss of RNA binding affinity, but increases the ATPase activity by 2-fold (14). LlaBIII^R564A^ was an active ATPase but was slower at DNA translocation, suggesting a partial decoupling of the translocase and ATPase. This conclusion is consistent with the role of motif V proposed in case of SF2 ATPases such as chromatin remodeler SWI/SNF (12). The arginine, which in LlaBIII is located on the path traversed by the DNA (Figure 1B), is a conserved residue of motif V of SF2 ATPases, and is found to interact with the phosphate backbone of the bound DNA in many of them (26,27). The interaction is expected to grip the DNA during translocation. The ATPase active site is located spatially away from DNA binding surface (4), and, hence, we think that LlaBIII-R564 may not be directly involved in ATP hydrolysis.

Loss of interaction with the DNA due to mutation of LlaBIII-R564 would have affected traction, and hence reduced the rate of translocation by LlaBIII^R564A^. Based on studies of DNA helicases and translocases, it is thought that DNA translocation requires interaction between amino acids on the DNA binding surface of the ATPase with DNA. In general, these interactions grip the DNA and pull it, and the grip on the DNA is released when the interactions are broken. Formation and disruption of the interactions made by the ATPase domain with DNA is driven by the conformational changes in the ATPase domain upon ATP binding and hydrolysis. Loss of one or more of these critical interactions loosen the grip on the DNA and can affect DNA translocation, even if ATP binding and hydrolysis by the ATPase is not affected.

In Type ISP RM enzymes, unlike in many other SF2 ATPases (13,15–16), motif III is masked from interacting with the substrate by the β-hairpin loop that is positioned adjacent to the motif (Supplementary Figure S13). As a consequence, motif III is not expected to be involved in DNA translocation *via* direct interaction with the substrate. But being adjacent to the β-hairpin loop, residues of motif III may be affected by the changes in the loop on DNA binding. We find that the mutation of Thr376 of motif III decreases ATPase activity, which decreases the rate of translocation by LlaBIII^T376A^. However, unlike LlaBIII^R564A^, LlaBIII^T376A^ displayed a visible nucleolytic activity. This suggested the requirement of a threshold rate of translocation for the nuclease to be activated.

The slower rate of DNA translocation by LlaBIII^R564A^ hindered its single-strand nicking and dsDNA cleavage activities, which, however, was restored in cooperation with a WT enzyme, revealing the significance of the rate of translocation on the nucleolytic activity. It is possible that the slower translocating mutants upon convergence fail to achieve the cleavage-competent conformations. Unlike LlaBIII^T376A^ and LlaBIII^R564A^, we found that LlaBIII^ΔLoop^, which is an active ATPase but a completely inactive translocase, failed to cleave DNA on collision with a translocating WT enzyme. This suggests that the enzymes have to be translocation-competent and that both of them should have disengaged from their respective recognition sequence for DNA cleavage to occur. It is also possible that the slower translocating mutant LlaBIII^R564A^ fails to cleave DNA because they do not have enough traction on the DNA to push against each other and activate the nuclease upon head-to-head convergence.

It has previously been shown that an ATPase inactive mutant of LlaBIII, which remains statically bound to its target site, on colliding with a translocating WT enzyme nicks the DNA but fails to form a double-strand DNA break (20). Combined with the data from this study, we conclude that double-strand DNA break by Type ISP RM enzymes not only requires ATP hydrolysis but also translocation initiation. As Type ISP RM enzymes are a variant of Type I RM enzymes (28), we think that the activation of the nuclease of the ATP-dependent Type I RM enzymes would also be dependent on the efficiency of their translocation. In the case of Type III RM enzymes, the other class of ATP-dependent RM enzymes, ATP hydrolysis and interaction between partner proteins are essential for activation of the nuclease for DNA nicking and for double-strand DNA break (29). However, as the convergence of two Type III RM enzymes is driven by diffusion rather than active translocation (29–31), their nucleolytic activity will be independent of translocation-mediated activation and collision.

In conclusion, the present study of the Type ISP RM enzyme reveals that the enzymes translocation efficiency modulates its nuclease activity, and forms a platform for future studies of the molecular mechanism of translocation-coupled nuclease activation using single-molecule biophysics and structural biology.

## Supplementary Material

gkaa023_Supplemental_File
