# Supplementary material for: DNA-mediated coupling of ATPase, translocase and nuclease activities of a Type ISP restriction-modification enzyme
Source: Nucleic Acids Res. 2020 Jan 24;48(5):2594–603. doi: 10.1093/nar/gkaa023 (PMC7049714; doi:10.1093/nar/gkaa023)
Supplement: gkaa023_Supplemental_File [file gkaa023_supplemental_file.pdf]

Supplementary Information

DNA-mediated coupling of ATPase, translocase and nuclease activities of a Type ISP restriction-modification enzyme

Mahesh Kumar Chand, Vanessa Carle, K. G. Anuvind, Kayarat Saikrishnan*

Division of Biology, Indian Institute of Science Education and Research, Pune, 411008, India.

****Corresponding author***

email: saikrishnan@iiserpune.ac.in

Phone: +91 2025908047

Fax: +91 2025908186

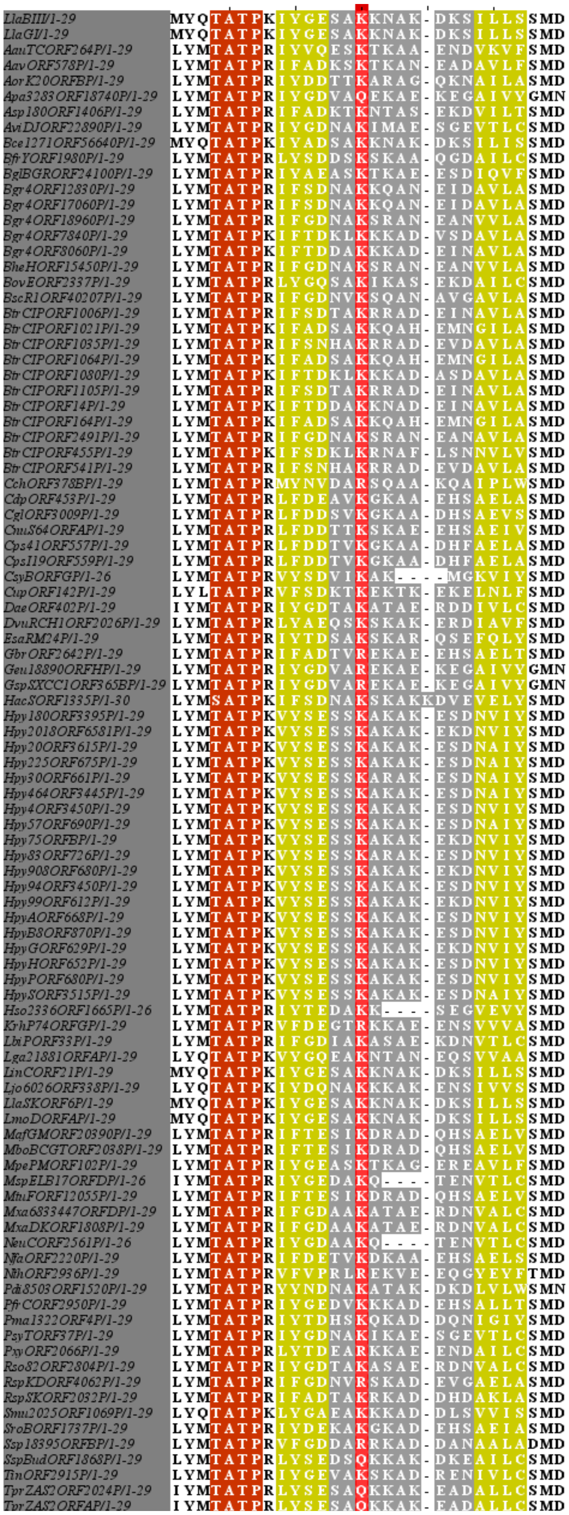

Supplementary figure 1: Amino acid sequence alignment of β-hairpin loop region in Type ISP R-M enzymes. The conserved lysine highlighted in red corresponds to LlaBIII-Lys385.

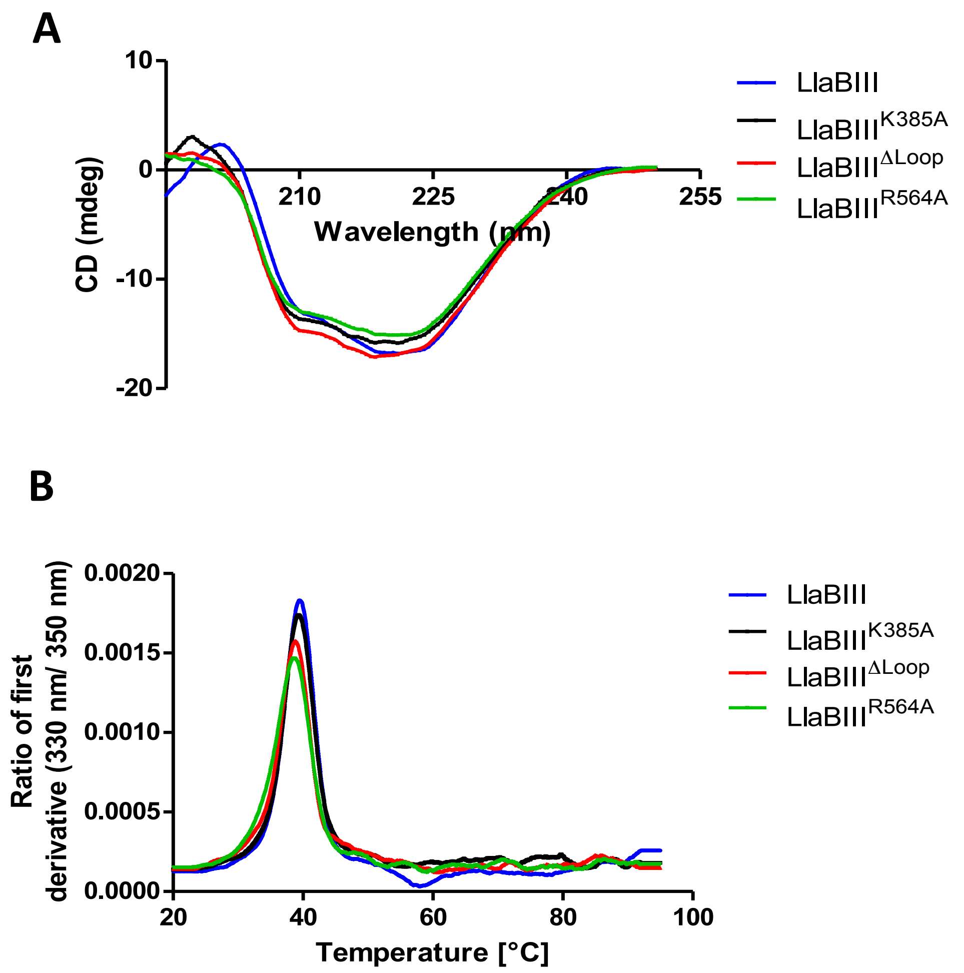

Supplementary figure 2: (A) Circular dichorism and **(B)** nanoDSF showing the conservation of secondary and tertiary structures of LlaBIII and its mutants.

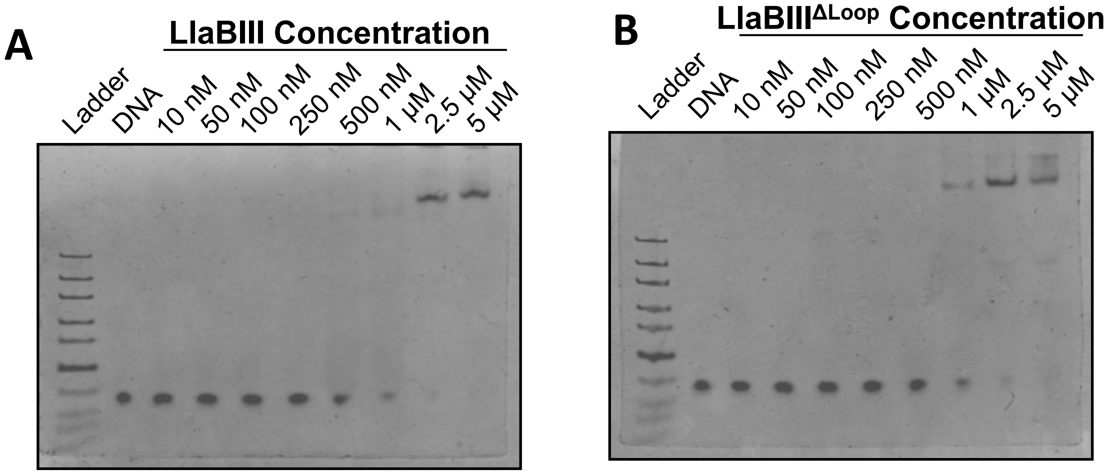

Supplementary figure 3: Electrophoretic mobility shift assay comparing the DNA binding affinities of **(A)** LlaBIII and **(B)** LlaBIII$^{\Delta Loop}$ for a 28 bp non-specific DNA.

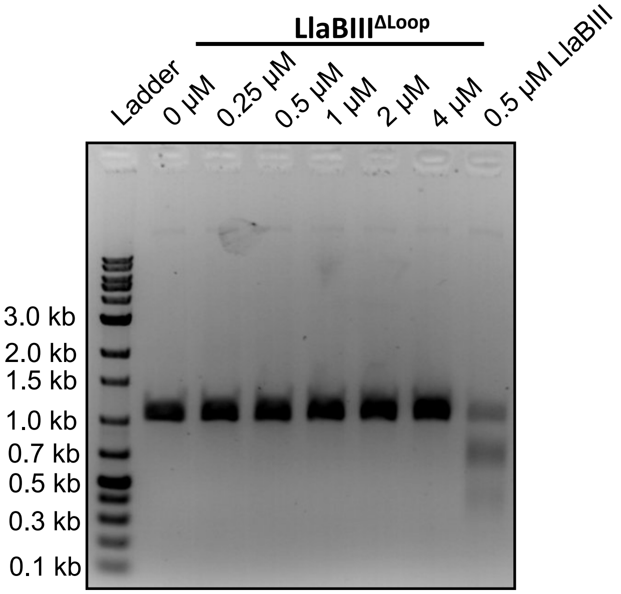

Supplementary figure 4: Concentration dependent DNA cleavage assay for LlaBIII$^{\Delta Loop}$ shows that this mutant is unable to cleave substrate DNA even at concentration of 4 µM. Last lane shows DNA cleavage by LlaBIII wild type at 500 nM concentration.

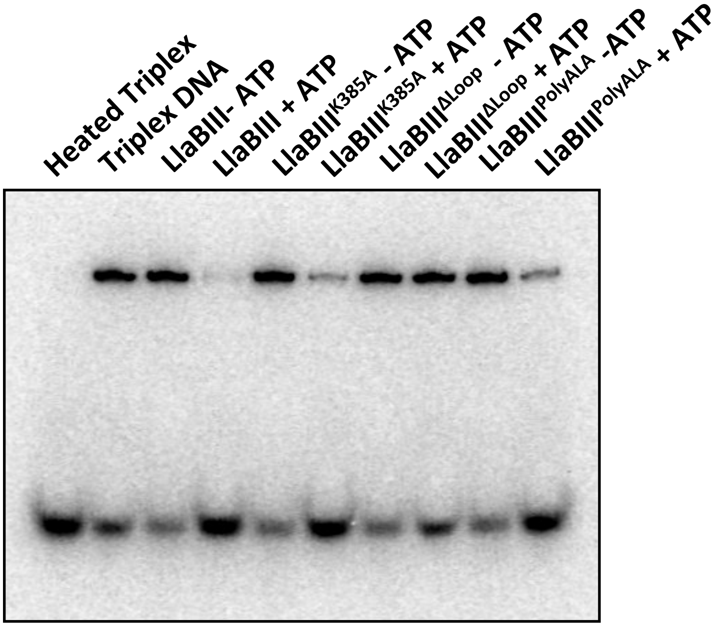

Supplementary figure 5: A representative gel of the triplex displacement assay showing the displacement of TFO by β-hairpin loop mutants in 10 minutes (see Figure 3B).

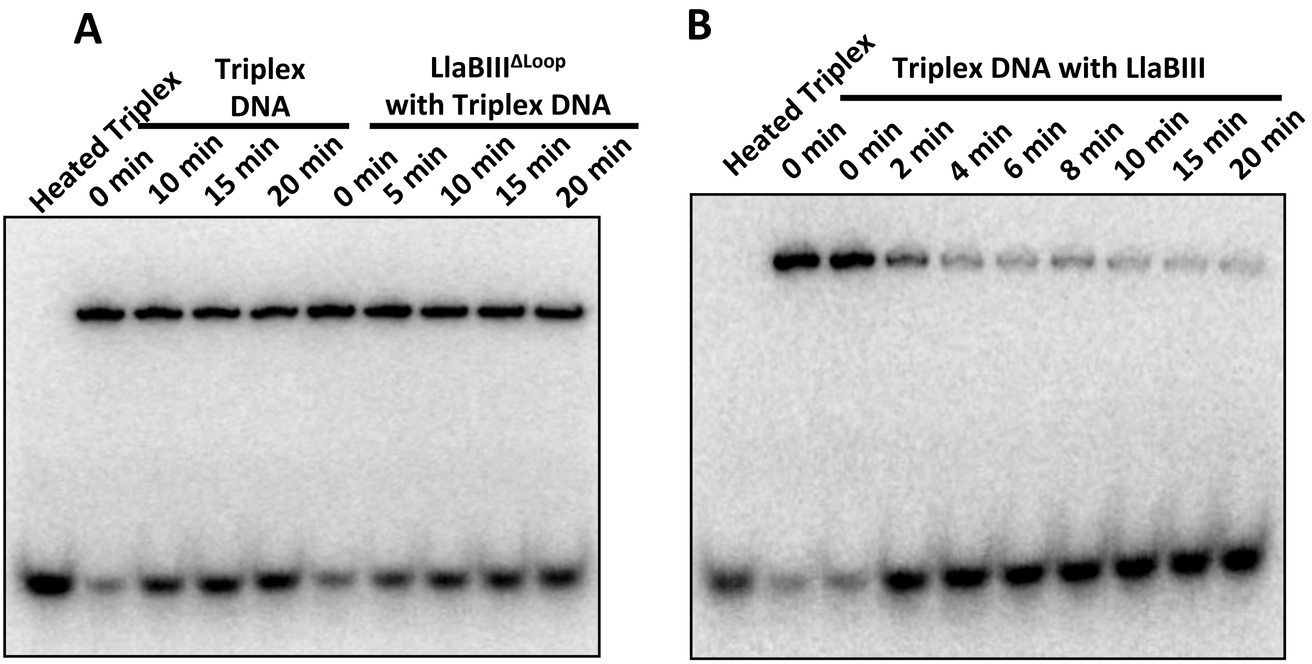

Supplementary figure 6: A representative gel of the triplex displacement assay showing time dependent displacement of TFO by LlaBIII and LlaBIII$^{\Delta Loop}$. (**A)** Triplex DNA control and LlaBIII$^{\Delta Loop}$ **(B)** LlaBIII. (See Figure 3C)

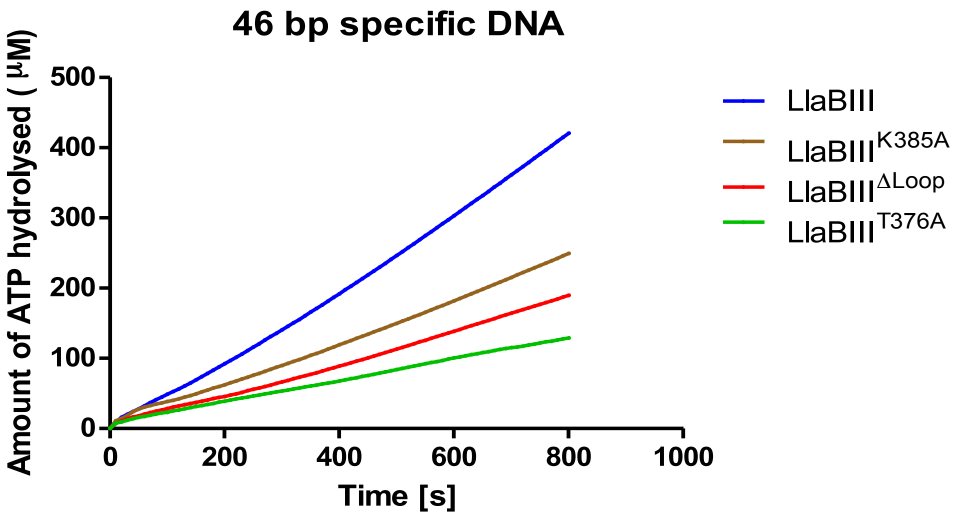

Supplementary figure 7: Comparison of the ATPase activity of LlaBIII^{T376A} with LlaBIII^{K385A} and LlaBIII$^{\Delta Loop}$ in presence of 46 bp specific DNA and 1 mM ATP carried out at 25^{0}C.

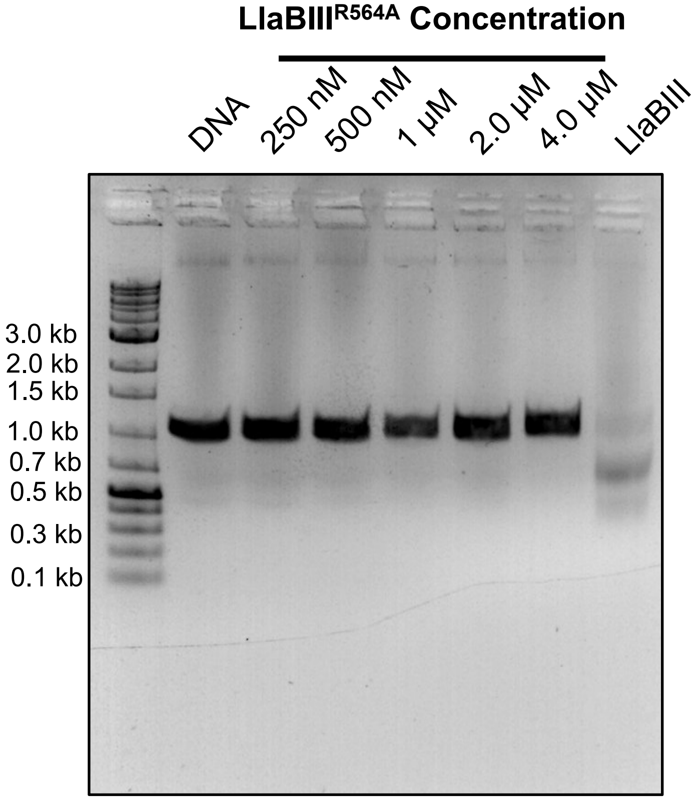

Supplementary figure 8: Concentration dependent DNA cleavage assay of LlaBIII^{R564A} shows that this mutant is unable to cleave substrate DNA even at concentration of 4 µM. Last lane shows DNA cleavage by WT LlaBIII at 500 nM concentration.

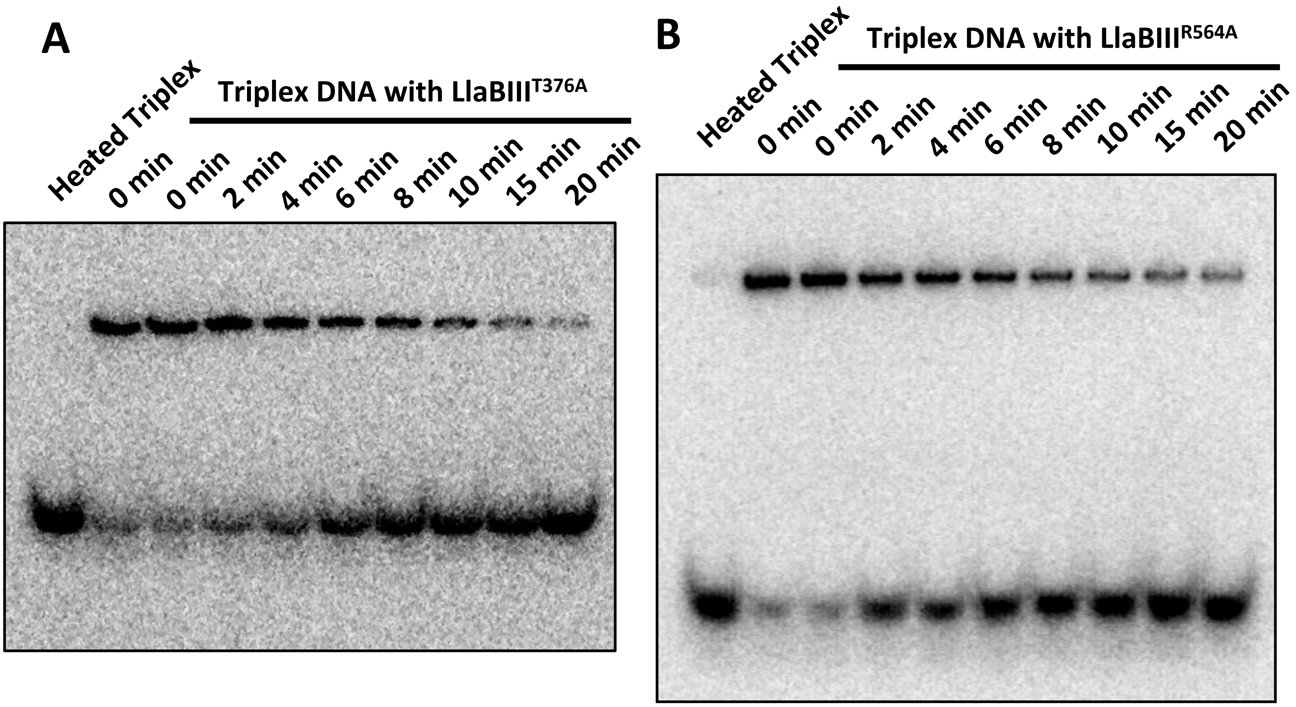

Supplementary figure 9: A representative gel of the triplex displacement assay showing time dependent displacement of TFO by (**A)** LlaBIII^{T376A} and **(B)** LlaBIII^{R564A} (see Figure 4E).

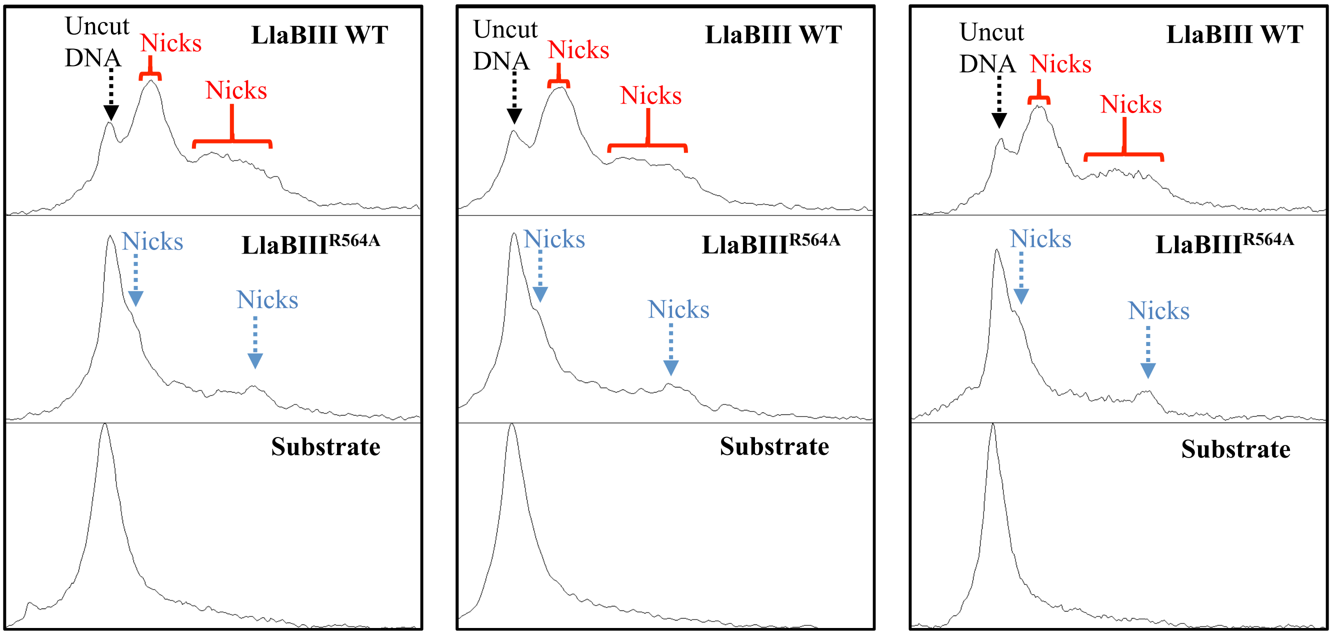

Supplementary figure 10: DNA nicking by LlaBIII and LlaBIII^{R564A}. Densitometry of the urea-formamide PAGE showing faint nicking by LlaBIII^{R564A} whereas LlaBIII nicks DNA with high efficiency. Black arrow shows the single-stranded uncut DNA. The red brackets shows the nicked product by LlaBIII whereas the blue arrows shows the nicked product by LlaBIII^{R564A}.

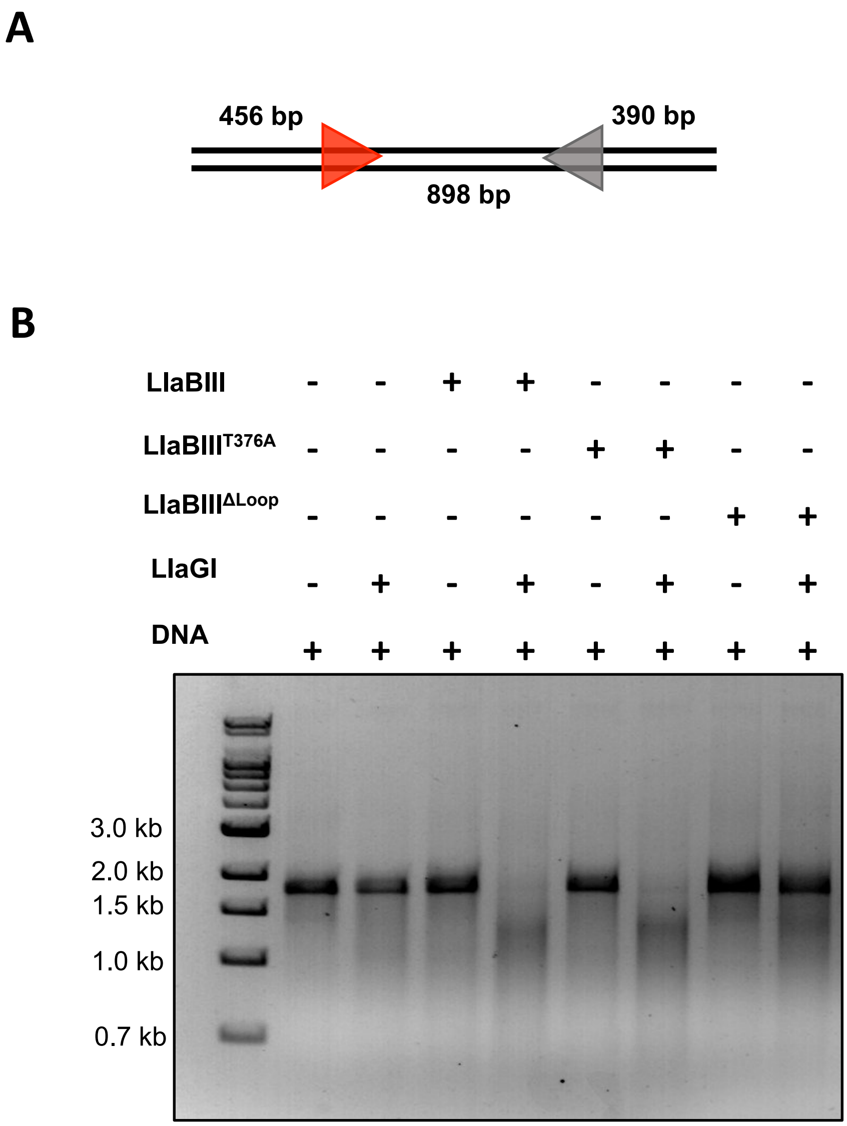

Supplementary figure 11: Heterologous cooperation assay. (A) Schematic of the DNA substrate used with LlaBIII site in red and the LlaGI site in grey. **(B)** DNA cleavage by LlaBIII or its mutants in cooperation with LlaGI. 500 nM of each enzyme was incubated with 10 nM of DNA and 4 mM of ATP for 30 minutes at 25 ^{0}C.

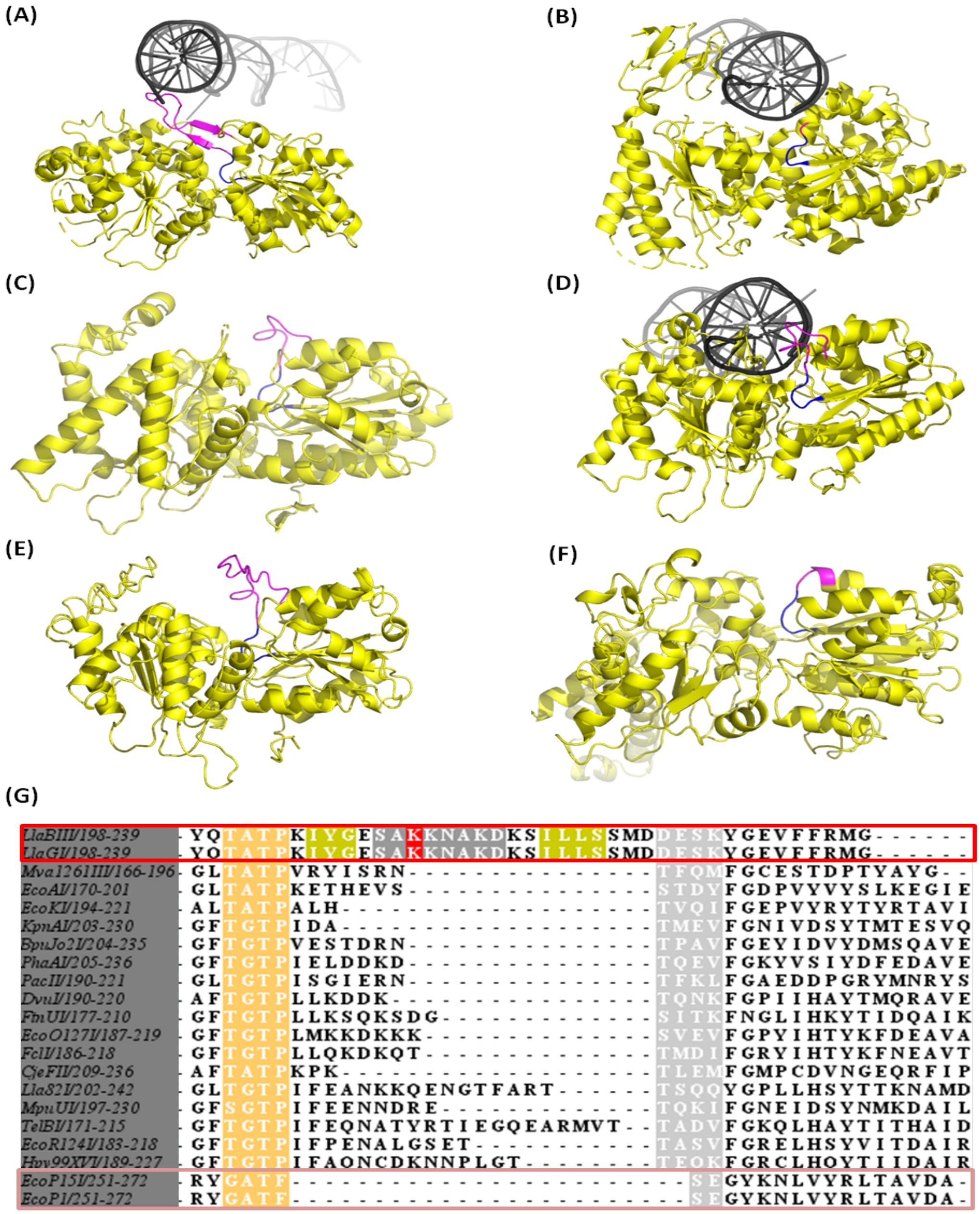

Supplementary figure 12: Structural comparison of the ATPase domains of (A) DNA-bound LlaBIII, (B) DNA-bound Type III RM enzyme EcoP15I (32), (C) Type I RM enzyme EcoR124I (33), (D) EcoR124I with a modeled DNA indicating the possibility of the interaction of hairpin loop (magenta) with the DNA, (E) Type I RM enzyme TelBI modeled using I-TASSER (34) and (F) EcoKI modeled using I-TASSER. The position of the β-hairpin loop is colored magenta. Blue color represents motif III. The β-hairpin loop appears to be present in EcoR124I and TelBI, but is absent in EcoKI and EcoP15I. (G) Sequence alignment of a part of the helicase domain of Type ISP (LlaBIII and LlaGI), Type I and Type III (EcoP15I and EcoPI) indicating the possible presence of an equivalent loop in Type I RM enzymes.

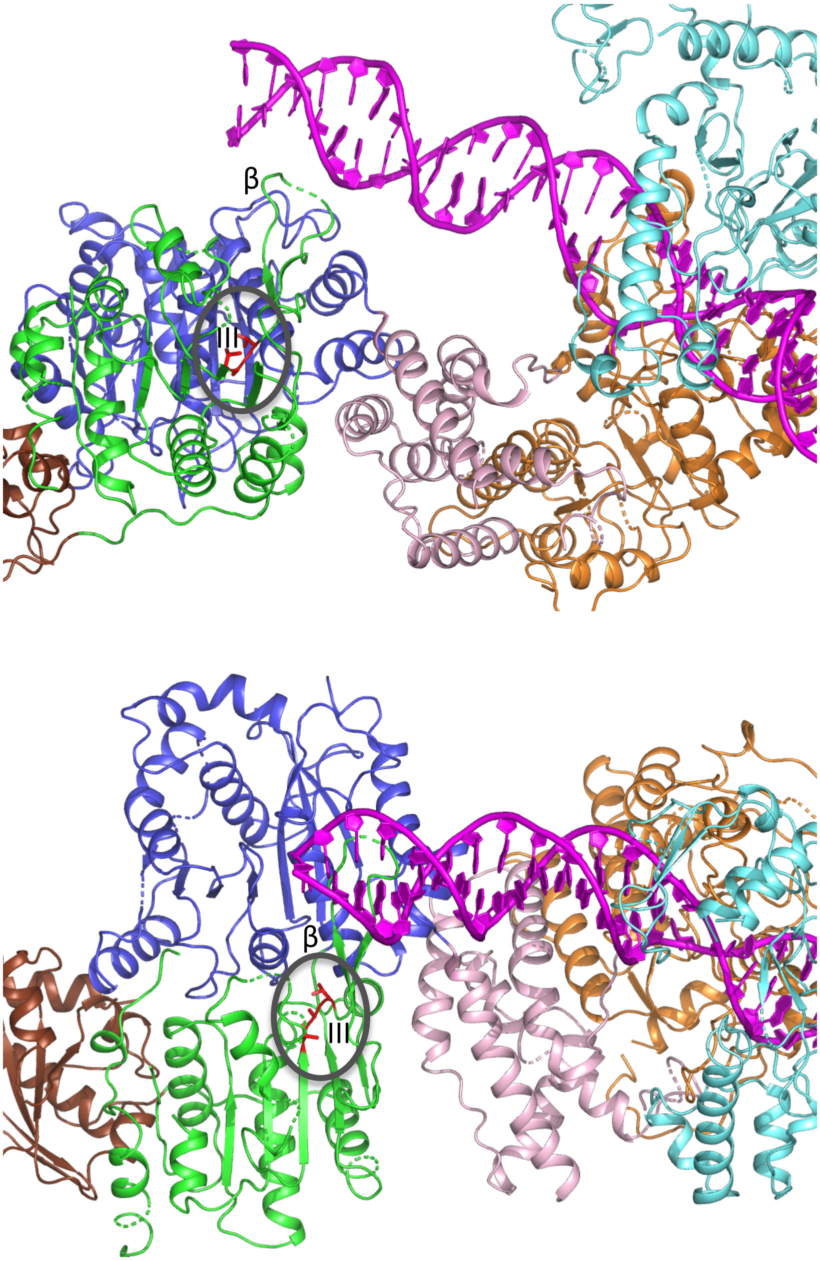

Supplementary figure 13: Structure of LlaBIII-DNA complex highlighting the position of motif III (red) with respect to the DNA and β-hairpin loop.

Supplementary Table 1: DNA oligomers used for the study.

Primer	*Sequence*
LB-pRSF-F	ACTTTAATAAGGAGATATACCATGGTGGCATTTTGGAAGGAATG
LB-pRSF-R	CGCAGCAGCGGTTTCTTTACCAGACTCGAGTTATAGTCCCTGTACTACTCTTG
K385A-R	GATACTCTTGTCTTTGGCATTTTTTGCCGCACTTTCCCCATATATCTTTGG
ΔLoop-2G-F	CACCAAAGATATATGGGGAAGGTGGTAAGAGTATCTTAC
ΔLoop-2G-R	GATGAAAGTAAGATACTCTTACCACCTTCCCCATATATC
PolyALA-R	GATACTCTTGTCTGCGGCAGCTGCTGCCGCAGCTTCCCCATATATC
T376A-R	CTTTCCCCATATATCTTTGGTGCAGCCGTTTGGTACATTC
R564A-R	CGATTCCTTCCGTTAAGAAGGCGACATTAGAAACAATTCG
LB-1074-F	GCAACGGACGCTCGCTGATCCAG
LB-1074-R	CCATCGCTTGGGAGACGGGGTTTTG
LJ1HISF	GAAGGAGATATACATATGGGTAAAATCGTCCTGCC
LJ1HISR1	GATGATGATGATGGGATCCTTATTCTTCCGTGGAC
200bp_FP	CTGTATGAAGCCCTGCAGAAC
1439bp_RP	TCTATTAATTGTTGCCGGGAAGC
Oligo23-F	TTAGCTAATAGACTGAGCCGAGG
Oligo23-R	TCCTCGGCTCAGTCTATTAGCTA
Oligo28-F	GCTCTAGCTAATAGACTGAGCCGAGGTG
Oligo28-R	CACCTCGGCTCAGTCTATTAGCTAGAGC
Oligo32-F	GCCTGCTCTAGCTAATAGACTGAGCCGAGGTG
Oligo32-R	CACCTCGGCTCAGTCTATTAGCTAGAGCAGGC
Oligo33-F	GACCTGCTCTAGCTAATAGACTGAGCCGAGGTG
Oligo33-R	CACCTCGGCTCAGTCTATTAGCTAGAGCAGGTC
Oligo34-F	GTACCTGCTCTAGCTAATAGACTGAGCCGAGGTG
Oligo34-R	CACCTCGGCTCAGTCTATTAGCTAGAGCAGGTAC
Oligo35-F	GTCACCTGCTCTAGCTAATAGACTGAGCCGAGGTG
Oligo35-R	CACCTCGGCTCAGTCTATTAGCTAGAGCAGGTGAC
Oligo46-F	GTCTTATGCAGGTCACCTGCTCTAGCTAATAGACTGAGCCGAGGTG

Oligo46-R	CACCTCGGCTCAGTCTATTAGCTAGAGCAGGTGACCTGCATAAGAC
TFO_1	TTCTTTTCTTTCTTCTTTCTTT
TFO_3	TTCTTTCTTTTCTTTCTTTCTT
NSP40-F	GTACTCAGCAGTATCCTGTATGCTACGTATTGCTATCGTG
NSP40-R	CACGATAGCAATACGTAGCATACAGGATACTGCTGAGTAC

Supplementary References:

32. Gupta, Y.K., Chan, S.H., Xu, S.Y. and Aggarwal, A.K. (2015) Structural basis of asymmetric DNA methylation and ATP-triggered long-range diffusion by EcoP15I. *Nature communications*, **6**, 7363.
33. Lapkouski, M., Panjikar, S., Janscak, P., Smatanova, I.K., Carey, J., Ettrich, R. and Csefalvay, E. (2009) Structure of the motor subunit of type I restriction-modification complex EcoR124I. *Nature structural & molecular biology*, **16**, 94-95.
34. Yang, J., Yan, R., Roy, A., Xu, D., Poisson, J. and Zhang, Y. (2015) The I-TASSER Suite: protein structure and function prediction. *Nature methods*, **12**, 7-8.
